# Editorial: Deciphering cell-cell interactions in triple-negative breast cancer

**DOI:** 10.3389/fimmu.2025.1766856

**Published:** 2026-01-07

**Authors:** Xue Yang, Lvyun Zhu, Xingrui Li, Yaying Du

**Affiliations:** 1 Department of Thyroid and Breast Surgery, Tongji Hospital, Tongji Medical College, Huazhong University of Science and Technology (HUST), Wuhan, Hubei, China; 2 Laboratory of Thyroid and Breast Surgery, Tongji Hospital, Tongji Medical College, Huazhong University of Science and Technology (HUST), Wuhan, Hubei, China; 3 Laboratory of General Surgery, Tongji Hospital, Tongji Medical College, Huazhong University of Science and Technology (HUST), Wuhan, Hubei, China; 4 College of Science, National University of Defense Technology, Changsha, Hunan, China

**Keywords:** cell-cell interaction, single-cell RNA sequencing, spatial transcriptomics, triple negative breast cancer, tumor immune microenvironment

Triple-negative breast cancer (TNBC) remains one of the most challenging subtypes of breast cancer. Lacking estrogen receptor (ER), progesterone receptor (PR), and human epidermal growth factor receptor 2 (HER2) as therapeutic targets, it presents a highly aggressive biological profile at diagnosis (1). With higher early distant recurrence rates and poor five-year survival, TNBC stands as a top priority for breakthroughs in precision oncology (2). While extensive studies have explored its molecular subtypes, genetic traits, and signaling pathways, the roots of its invasiveness, immune evasion, and treatment resistance lie in the intricate network of cell–cell interactions (CCIs) (3). Critical evidence shows that even within the same molecular subtype, tumor microenvironment (TME) variations drastically impact prognosis and immunotherapy response, elevating CCIs from a secondary factor to the core of TNBC research (4, 5).

TNBC’s heterogeneity is reflected not only in dynamic state shifts within tumor cells but also in the multi-layered ecosystem formed by crosstalk between tumor, immune, stromal, and endothelial cells (6, 7). Traditional histology and bulk RNA sequencing fail to capture these dynamic relationships, yielding only “averaged” signals that mask contextual details. However, the rapid advancement of single-cell RNA sequencing (scRNA-seq) and spatial transcriptomics (ST) has transformed the field (8). These technologies enable researchers to penetrate tissue layers, treat cells as interconnected nodes, and map their interactions across spatial and temporal axes (9, 10). The Research Topic *“Deciphering Cell-Cell Interactions in Triple-Negative Breast Cancer”* leverages this technological revolution to integrate cutting-edge research, deepen understanding of TNBC CCIs, and drive therapeutic innovation.

Within a single TNBC tumor, multiple molecular subtypes, proliferative states, and stem cell populations coexist, while immune-stromal ecosystems exhibit profound complexity. Single-cell and spatial omics confirm that cellular composition and function vary drastically across tumor regions (10). Early studies identified a layered spatial architecture: the tumor core, immune exclusion zone, and immune infiltration front (11, 12). Each has unique cellular compositions and interaction patterns. Here, tumor-associated macrophages (TAMs), regulatory T cells (Tregs), tumor-associated neutrophils (TANs), and cancer-associated fibroblasts (CAFs) collaborate to suppress anti-tumor immunity via cytokines, chemokines, and metabolic reprogramming (4). Response to anti-PD-1/PD-L1 immunotherapy often depends on the spatial arrangement of CD8^+^ T cells, Tregs, myeloid cells, and CAFs, as well as the activity of ligand–receptor axes like CXCL/CXCR and IL1/IL1R (5). Deciphering these tumor-immune-stromal interactions is thus pivotal to improving immunotherapy efficacy.

## Unveiling tumor-immune interaction networks: from evasion to precision intervention

A review in this Research Topic constructs a comprehensive framework for TNBC immune evasion, emphasizing synergistic immunosuppression among tumor, myeloid, and lymphoid cells (Garcia et al.). The TNBC immune microenvironment is defined by myeloid cell enrichment, T cell exhaustion, and sustained inflammatory signaling. Tumor-associated macrophages, neutrophils, and myeloid-derived suppressor cells (MDSCs) dominate the TME, impairing adaptive immunity through IL-10, TGF-β, ROS, and ARG1. Concurrently, tumor cells evade surveillance by downregulating MHC-I and upregulating PD-L1. These mechanisms interconnect in a network of signaling axes, feedback loops, and differentiation trajectories.

Another original study uses integrated single-cell analysis to position TANs as key network nodes and identifies core pro-tumor genes (Li et al.). Functional validation confirms TAN-driven interaction axes as promising therapeutic targets. These findings underscore the multicellular nature of TNBC’s immunosuppressive microenvironment and provide new molecular entry points for precision intervention.

## Single-cell and spatial omics: mapping CCI landscapes

The review on scRNA-seq and ST in this Research Topic reveals that TNBC CCI complexity stems from both cell type diversity and spatial organization (Xin et al.). Spatial analyses show Tregs and M2-TAMs form immunosuppressive “hotspots” in the tumor core, while activated CD8^+^ T cells localize to the periphery. Subsets like ANXA3^+^ epithelial cells suppress T cells via BTLA-TNFRSF14, and the TAM-tumor IL1α-IL1R1 axis drives EMT through positive feedback, enhancing invasion.

These discoveries illuminate CCI spatial specificity, validating the “structure-signaling-function” causal chain inaccessible to traditional models. Critically, spatial omics enable the identification of key nodes driving tumor ecology.

## From tissue to body fluids: systemic CCI signatures

TNBC CCIs extend beyond the local TME to generate detectable systemic signatures. A clinical study in this Research Topic introduces the pan-immune-inflammation value (PIV), derived from peripheral blood neutrophil, monocyte, platelet, and lymphocyte ratios, as a robust prognostic tool for post-operative TNBC patients. High PIV correlates with enhanced systemic inflammation and impaired anti-tumor immunity, mirroring the myeloid-predominant, lymphoid-depleted CCI features in the TNBC microenvironment. This highlights the need for CCI research to bridge intra-tissue interactions with accessible biomarkers, enabling non-invasive monitoring of disease and treatment response (Qi et al.).

## Challenges and future directions

Technical hurdles persist. scRNA-seq batch effects, cell capture biases, and limited sensitivity for rare populations remain unresolved (13). High-resolution ST sacrifices coverage, complicating “whole-tumor + single-cell” analysis, while CCI inference tools (CellPhoneDB, CellChat, NicheNet) yield inconsistent results, requiring robust follow-up validation (14, 15).

Clinically, most omics studies remain exploratory, with few biomarkers integrated into decision-making. Distinguishing “driver” (targetable) from “passenger” (incidental) CCIs lacks functional validation, and compressing complex CCI atlases into actionable test panels remains unmet. Promisingly, some studies use ST to link spatial gene expression/drug sensitivity gradients to ligand-receptor patterns for treatment prediction (16–18). In TNBC, researchers are mapping pre- and post-treatment TME remodeling via single-cell/spatial omics to identify response-related CCIs, laying the groundwork for personalized therapy (19–21).

As technology evolves and interdisciplinary collaborations expand, decoding TNBC CCIs is poised to unlock new therapeutic avenues. Integrating multi-omics, functional validation, and clinical translation will move beyond one-size-fits-all approaches to precision medicine tailored to each patient’s unique tumor ecosystem, offering hope for overcoming TNBC’s aggressiveness.
